# Chronic fatigue syndrome/myalgic encephalomyelitis in children aged 5 to 11 years: A qualitative study

**DOI:** 10.1177/1359104520964528

**Published:** 2020-10-22

**Authors:** Amberly Brigden, Alison Shaw, Emma Anderson, Esther Crawley

**Affiliations:** Population Health Sciences, Bristol Medical School, University of Bristol, Bristol, UK

**Keywords:** CFS/ME, myalgic encephalomyelitis, chronic fatigue syndrome, paediatrics, qualitative research methods, intervention development

## Abstract

Treatments for paediatric chronic fatigue syndrome/myalgic encephalomyelitis (CFS/ME) have not been designed or evaluated for younger children (5–11-years). The development of a complex intervention for this population requires an in-depth understanding of the perspectives and psychosocial context of children and families. Children with CFS/ME (5–11-years) and their families were recruited from a specialist CFS/ME service, and interviewed using semi-structured topic guides. Data were analysed thematically. Twenty-two participants were interviewed; eight parents, two children (aged nine and ten) and six parent-child dyads (aged 5–11-years). Theme 1: CFS/ME in younger children is complex and disabling. Theme 2: Children aged eight and over (in comparison to those under eight) were more able to describe their illness, engage in clinical consultation, understand diagnosis and self-manage. Theme 3: Parents of children under eight took full responsibility for their child’s treatment. As children got older, this increasingly became a joint effort between the parent and child. Parents felt unsupported in their caring role. Clinicians should consider different treatment approaches for children under eight, focusing on: parent-only clinical sessions, training parents to deliver treatment, and increasing support for parents. Children over eight may benefit from tools to help them understand diagnosis, treatment and aids for self-management.

## Introduction

Paediatric Chronic Fatigue Syndrome or Myalgic Encephalomyelitis (CFS/ME) is characterised by severe and debilitating fatigue which typically increases after exertion with a range of symptoms including pain, sleep disturbance and cognitive dysfunction (NICE 2007). It is relatively common, with approximately 0.1% to 2% of adolescents affected by CFS/ME (Chalder et al., 2003; Crawley et al., 2011, 2012; Farmer et al., 2004; Nijhof et al., 2011; Rimes et al., 2007), and can lead to prolonged illness and disability (Kennedy et al., 2010). Approximately 12% of young people accessing paediatric CFS/ME services are below the age of 12 (Collin et al., 2015). Little is known about the epidemiology of CFS/ME in this younger age group (Collard & Murphy, 2019), but disability is high, with reduced school attendance and high levels of fatigue, anxiety, physical disability and pain (Davies & Crawley, 2008). The gender split is more balanced compared to adolescents (57% vs 74% female) (Collin et al., 2015), younger children are less likely to report cognitive dysfunction, problems with sleep or headaches, and more likely to present with recurrent sore throats, tender lymph nodes and dizziness (Collin et al., 2015).

Treatments recommended by National Institute for Health Care Excellence (NICE 2007) include Cognitive Behavioural Therapy (CBT) and behavioural approaches (Graded Exercise Therapy, GET, and activity management) (Brigden et al., 2017). All treatments offer advice on sleep, symptom management and co-morbidities such as mood disorders and pain. CBT, which has the strongest evidence base (Al-Haggar et al., 2006; Chalder et al., 2010; Lloyd et al., 2012; Nijhof et al., 2012; Stulemeijer et al., 2005), supports the young person to identify and change fatigue-related cognitions and gradually resume activities (Brigden et al., 2017). GET aims to stabilise physical activity levels, before gradually increasing activity at a manageable rate (White et al., 2007). Activity Management is a goal-oriented and person-centred approach which establishes a baseline for all activity (physical, cognitive and emotional), which is then increased (White et al., 2007).

These treatments have not been designed or evaluated for children aged 5 to 11 years; most treatment trials have recruited children aged ten or older (Al-Haggar et al., 2006; Chalder et al., 2010; Lloyd et al., 2012; Nijhof et al., 2012; Stulemeijer et al., 2005), with only one RCT recruiting children as young as eight (Brigden et al., 2016), and none that include children younger than eight. It is likely that these treatments need adapting to meet the specific developmental needs of this younger population.

Qualitative methods are recommended to inform intervention development/adaptation (Bartholomew et al., 2011; Campbell et al., 2007; Hoddinott, 2015; Wight et al., 2015). They can be used to gain a rich understanding of the individual’s experience of their health condition and provide insights into their needs for treatment (Corrrigan et al., 2006; Service NRD, 2014). Incorporating these views can lead to interventions that are more relevant, acceptable and usable (Yardley et al., 2015).

The study aimed to understand the perspectives and psycho-social context of younger children with CFS/ME (and their parents) to develop recommendations for adapting treatment for this younger age group. Interviews aimed to explore participants’ lived experience of CFS/ME, their experiences of receiving treatment within a specialist CFS/ME service and their views on how treatment could be adapted to better meet the needs of this younger age group.

## Methods

### Design

We undertook semi-structured qualitative interviews.

### Recruitment and sampling

Children and caregivers were recruited from:

- The ‘EXPLORER’ study was a longitudinal cohort study exploring the epidemiology of CFS/ME in younger children and the qualitative experiences of this population. Children were eligible for EXPLORER if they had a confirmed diagnosis of CFS/ME (using NICE guidelines (NICE, 2007)) and were aged between 5 and 11-years. Recruitment took place between February 2017 and February 2019.
- The ‘MAGENTA’ trial was an RCT evaluating the effectiveness and cost-effectiveness of GET versus activity management for young people with CFS/ME (Brigden et al., 2016). Young people were eligible if they had a confirmed diagnosis of CFS/ME (using NICE guidelines (NICE, 2007)) and were aged between 8 and 17 years. Young people were excluded if they were severely affected, if they were referred for CBT at their initial assessment, or if they were unable to attend clinical appointments. Recruitment took place between September 2015 and March 2018.

In each study, families could consent to take part in a qualitative interview. Purposeful sampling (Barbour, 2001) was used to identify a heterogeneous sample in terms of the child’s gender, age, school attendance and duration of illness. Throughout data collection and analysis, consideration was given to whether new interviews were adding additional insights for the purpose and goals of the study (pragmatic saturation) (Braun & Clarke, 2019) and whether there was enough quality data to answer the research questions (information power) (Malterud et al., 2015).

### Data collection

Families decided whether the child would be interviewed separately or in a dyad with their parent (Morgan et al., 2013). Families were interviewed at their preferred location (University, participants home or Skype (parents only)). Parent interviews were designed to last around 1 hour. The topic guide (see appendix) began with narrative interviewing techniques (Jovchelovitch & Bauer, 2000; Muylaert et al., 2014) to gather inductive and exploratory data on parental experiences of CFS/ME. The topic guide moved on to specific questions about assessment and treatment, and how these could be adapted for younger children.

The child interviews were designed to last for 30 minutes. Child interviews included arts-based and play methods, which can be effective with children across the developmental continuum (Coad, 2007). These are more engaging for children, more appropriate for their communication skills and can reduce the confronting and formal dynamic of a one-on-one interview with an adult. The development of the arts-based activities was informed by a range of sources including the study aims, existing literature (on child health and qualitative methods with younger children), consultation with two patient and public involvement groups, and our reflections on early interviews.

Activity 1: What is the child’s experience of CFS/ME? Children were encouraged to describe their experience through a body mapping exercise, a technique commonly used in qualitative research, particularly with children (de Jager et al., 2016). Children were asked ‘What does it feel like to have CFS/ME?’ (using the child’s terminology for CFS/ME), they were given the outline of a body and were encouraged to use pencils and stickers to illustrate their experiences. In addition to physical symptoms, it is important to understand the child’s experience of the psychological aspects of a chronic illness (Steinhauer et al., 1974). Therefore, children were also given ‘emoji’ cards as aids to communicate their emotional experiences. As the ability to experience and express different emotions develops with age (National Scientific Council on the Developing Child, 2004), the range of cards made available to the child was dependent on age (with older children being given the option of more complex emotions).

Activity 2: What are the child’s views on treatment and how this could be adapted to meet their needs? Children were presented with a visual ‘map’ of a medical consultation room, and they were encouraged to use Lego, stickers and pencils to illustrate who was in the treatment session and what happened. If children struggled with the free-recall nature of this activity, then it was supplemented with a card-sorting activity; children were shown images representing possible topics of conversation with their clinician (e.g. ‘we talked about an activity diary’). They were given the opportunity to pick the ones that they remembered discussing and were then asked follow-up questions to encourage them to expand upon this.

The arts-based activities were used as tools to prompt verbal accounts from the child participants. For each activity, the interviewer began with an open question based on the activity, with more specific planned follow-up prompts. The interviewer also asked spontaneous follow-up questions, to encourage participants to expand on certain subjects that they raised. These interviews were audio-recorded and transcribed. We also kept any images resulting from the arts-based activities, this included original drawings and photographs of the activities.

We ensured that transcripts and visual data were anonymised.

### Data analysis

Transcripts were imported into the qualitative data management software NVivo (NVivo Qualitative Data Analysis Software, 2012) and analysed thematically (Braun & Clarke, 2006, 2014). Analysis was ongoing and iterative, commencing soon after each interview. The lead researcher (AB) analysed all transcripts. Analysis was supported by experienced qualitative researchers (EA, AH) who independently coded a subset of transcripts (five in total). Researchers carried out systematic line-by-line descriptive coding, which was then grouped into broader themes

The data from children also included visual data from the arts-based activities. We analysed the transcripts and visual data simultaneously, whilst coding each transcript in NVivo. We reviewed the corresponding images to provide contextual information to the transcript. We considered the possible meanings of the images and how these fitted within developing themes.

## Results

### Participants

To guard against deductive disclosure (Kaiser, 2009) we do not provide the characteristics of individual participants, instead, we provide the aggerate characteristics of our sample. Twenty-two participants (across 14 families) were interviewed; eight parents, two children (aged 9 and 10) and six parent-child dyads. Considering the demographics of the index child, eight (57%) were female, five were aged between 5 and 7-years, nine were aged 8 to 11-years with a mean age of 8.5 years, all were white British. There was variation in school attendance (range 0–100%, mean 60%) and illness duration (range 9–63 months, mean 24 months).

Quotes are presented with a pseudonym and the age of the index child. We have used the age categories Key Stage One (KS1), corresponding to the ages 5 to 7 years, and Key Stage Two (KS2), corresponding to ages 8 to 11 years (Department for Education, 2013).

### Themes

#### Theme 1: CFS/ME in younger children is disabling and complex

There was a range of illness severity, but most families described CFS/ME as a persistent (‘*You realise it’s been going on and on*’, Emma’s parent, KS1) and disabling condition. It impacted the child’s hobbies, activities of daily living such as mobility (‘*really tired um and just struggling even to go up and down the stairs*’, Sophia’s parent, KS2), and in the majority of cases it affected schooling. Some families also described the impact on social activities and a desire for social support.

*I’d like to meet someone that has been through the same thing . . . so I don’t feel like I’m the only one cos I barely have met anyone that’s really the same age and knowing what this feels like. Like having to go to the hospitals and having appointments and things.* (Olivia, KS2)

Symptoms included significant fatigue

*absolutely shattered. . . we were waking for the first three days to eat and then he was back to sleep again. . . absolute really severe exhaustion* (Connor’s parent, KS2)

In addition symptoms were feeling ‘*weak*’ (Willow, KS2); disturbed and unrefreshing sleep (‘*I just feel very tired no matter how well I’ve slept*’, Willow, KS2); digestive symptoms (‘*stomach pain um, IBS like type symptoms*’, Chloe’s parent, KS2); muscle and joint pain (‘*a lot of joint pain. Ankles, feet, knees*’, Chloe’s parent, KS2); ‘*sore throats*’ (Lyra’s parent, KS2) and headaches. Families also described emotional difficulties secondary to the physical disability (‘*I used to cry in the morning because I felt so tired*’, Sophia, KS2; ‘*he’ll cry, pull at his eyes and almost like hyperventilate*’, Levi’s parent, KS2). Families also observed fluctuating symptoms (‘*she goes through phases of being exhausted and then phases where she seems fine*’, Emma’s parent, KS1) and post-exertional malaise (Parslow et al., 2018)

*he’d play football for two hours . . . two days later he was just flat on the sofa. He couldn’t do anything to the point where my husband had to carry him upstairs to his bedroom.* (Ethan’s parent, KS2)

Parents talked about the complexity of cause, onset and maintenance of CFS/ME. Most talked about a viral trigger, with psycho-social factors contributing to the development and/or maintenance of the illness alongside the infection. One parent described a complex combination of genetic predisposition, immune dysfunction, dietary allergies, psycho-social stress and poor gut health. Some parents wanted more in-depth explanations of this complex diagnosis.

I *had ME before I was pregnant, it can be genetic . . . my theory is that she’s had so many viruses that it has compromised her immune system . . . she’s picked the ME up but also . . . she’s allergic to dairy, she has very funny stomach and probably a leaky gut . . . and there’s a huge link there, between CFS and the gut. . . and her grandad died when she was four and I think that has had an impact. . . she was very stressed, very distraught. . . I think obviously anything emotional impacts on the physical . . . it could have been linked to that shock as well.* (Ava’s parent, KS1)

Parents commonly talked about the added complexity of their child having co-morbid conditions. Some felt the CFS/ME service did not always address this complexity, and that the care for CFS/ME and other conditions was disjointed. Some wanted more medical management others wanted more psychological input

*I was quite surprised there wasn’t more in the way of psychological support offered. . . because of all the anxiety and low mood that she’s had hand in hand with it all.* (Sophia’s parent, KS2).

#### Theme 2: The child’s capacity to understand and manage a chronic health condition

Comparing younger children (below 7 years) to the older, families described differences in the child’s ability to engage in clinical consultations and understand and manage their condition. This variation across the developmental range is considered throughout the results. Whilst we acknowledge a spectrum rather than a dichotomy, to illustrate developmental differences we have chosen to refer to two age groups: 5 to 7-years (KS1) and 8 to 11-years (KS2).

##### Engaging in consultations, describing experiences and understanding diagnosis and treatment

**Figure 1.**
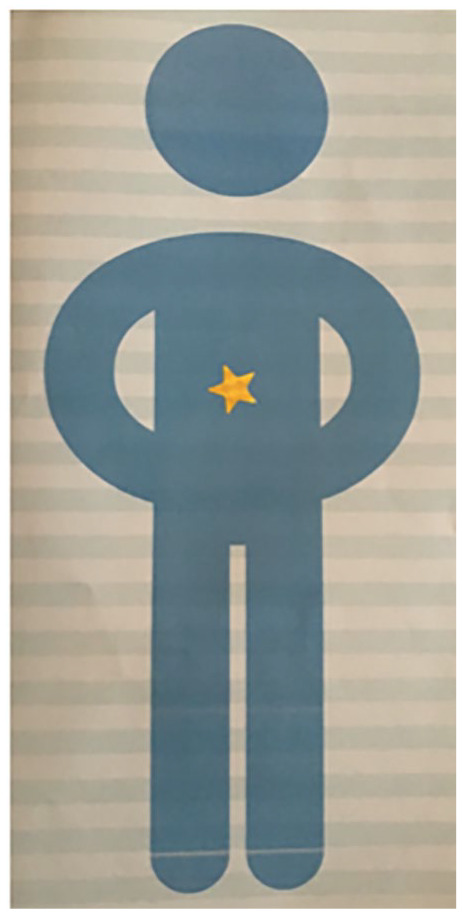
A younger child’s description of their CFS/ME experience (Leo, KS1). Child: *um no* Mum: *you get tummy aches don’t you* Child: *yes* Mum: *put a sticker down on there*

Many parents of KS1 children reported that their child struggled to focus and engage during clinical sessions; ‘*switched-off. . . a bit bored*’ (Henry’s parent, KS1), and that most dialogue occurred between clinician and parent. Some parents reported children were ‘*fed up of hospital appointments*’ (Jacob’s parent, KS1) and that appointments sometimes increased their child’s fatigue symptoms. Parents also reported that younger children found it hard to report symptoms. An example of a younger child’s description of their experience is illustrated in Figure 1.

*As an older child it would be easier to recognise because they can talk about it . . . you’re asking a* [young child] *how they feel they haven’t got a clue, it’s, ‘I feel ill.’* (Zoe’s parent, KS1)

Parents of KS1 children felt medical terminology such as ‘Chronic fatigue syndrome’ or ‘myalgic encephalomyelitis’ and resources for treatment were ‘*not appropriate for her age at all*’ (Emma’s parent, KS1). They preferred simple terms and explanations that focused on the symptoms of fatigue and helping the child to understand the need to reduce activity. The two KS1 children couldn’t remember or appeared not to understand the description of the illness their clinician had provided, such as the analogy of fatigue being like a battery, and were unsure about aspects of treatment such as medication ‘*I don’t know what the tablets are for*’ (Leo, KS1).

Interviewer: *Can you remember what the doctor said at that appointment?*

Parent: *she did explain to you about your. . .like your tiredness . . .. Can you remember what she said about you having a battery inside you?*

Child: *That’s funny*

Parent: *Yes, and how did she explain it to you?*

Child: *Can’t remember.*

Parent: *What did she say about if your battery gets really flat?*

Child: *I didn’t have a battery.*

Parent: *And that was what you kept saying to her.*

*(Leo and his parent, KS1)*

**Figure 2.**
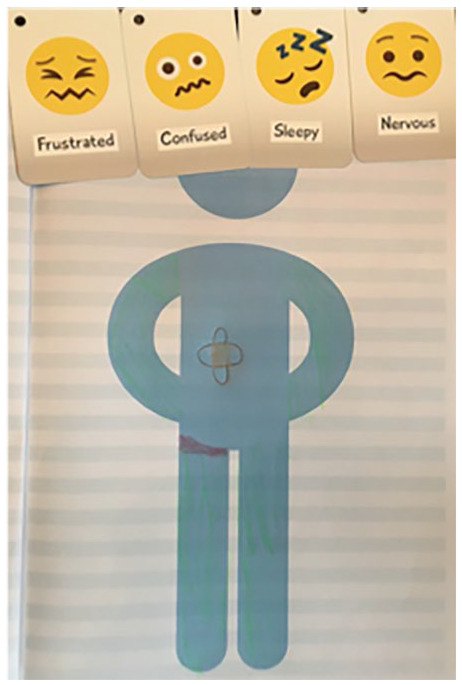
An older child’s description of their CFS/ME experience (Lyra, KS2). *I used to get this thing um that we used to call the leg thing which is where like it really hurts if like on my thigh, on my right thigh. . .. It really hurts and, and I like can’t move it. Um, sometimes if I walk in a weird way it’ll um, if I um like walk too much. I used to get it um really badly . . .Sometimes um I feel a bit sick like in my stomach um and I feel ill because um, I don’t know, I just feel sick sometimes when um, like I get really tired and um, just feel sick um yeah. . .. when I exercise a, a lot sometimes I will, I will feel quite tired. . . I feel nervous because like I know that I am going to get tired and I’ll feel sick. . . I am scared because obviously feeling sick doesn’t feel good so I try not to do too much because I’m scared that will happen. . . when I’m at school I feel kind of scared to tell people, so I don’t normally tell the teacher.* The participant coloured in the arms and legs green to represent heaviness and tiredness in limbs. The red line represented a pain in the thigh. The cross on the stomach represented nausea.

KS2 children were more active contributors to clinical consultations. Parents of these children talked about the ‘*benefits from* [child] *being involved at least partially*’ (Ethan’s parent, KS2). Families felt it was important for the child to express their own thoughts and feelings; parents didn’t want ‘*to presume how* [child] *feels’* (Ethan’s parent, KS2) and children felt *‘sometimes my mum thinks different things to what I think so I kind of in one way have to* [attend]*’* (Chloe, KS2). Some KS2 children independently provided richer accounts of physical symptoms and emotional experience (see Figure 2) using casual language such as ‘*feel like bleurgh*’ (Lyra, KS2) ‘*feel very slouchy*’ (Willow, KS2) ‘*I feel my eyes drooping down*’ (Sophia, KS2) and ‘*my brain starts to hurt*’ (Sophia, KS2). Parents of KS2 children felt ‘*from eight they tend to understand more*’ (Jacob’s parent, KS2) about explanations of CFS/ME and treatment, and this was aided by visuals and analogies

*she got a diagram out, it was like the ups and downs* [boom- bust] *visually when he looked at it, he understood that a lot more.* (Jacob’s parent, KS2)

During the interview, some KS2 children provided accounts which considered biological mechanisms. Most provided brief illness explanations, focusing on a lack of energy and feeling tired. They found medical terminology challenging and reported ‘*if I didn’t get anything I asked mum*’ (Sophia, KS2)

*it was like how tiredness works and how your energy works. . . And I don’t produce as much like melatonin what gets you to sleep . . . there were weird names for each thing.* (Willow, KS2)

KS2 children wanted more engaging and child-friendly resources to explain the condition; ‘*magazines and comics*. . .*a quiz*. . . *fun chronic fatigue games*’ (Connor, KS2) and ‘*videos*’ (Willow, KS2).

##### Engaging in self-management

CFS/ME management involves monitoring, recording and regulating behaviours in accordance with a treatment plan, including sleep and activity. Parents of KS1 children felt their child ‘*haven’t got the capability*’ (Ava’s parent, KS1) to self-manage. Parents of KS2 children felt that children ‘*understood*’ treatment principles, such as regulating sleep and activity, but did not have the maturity for self-regulation, ‘*left to self-manage she over did it*’ (Chloe’s parent, KS2). Self-control, delaying gratification, imposing restrictions on themselves and weighing-up consequences (e.g. worsening symptoms) were thought to be beyond the child’s developmental capabilities.

*it’s challenging with younger children . . . With an adult, there’s amount of reasoning and being rational that you can’t expect from a ten-year-old* (Ethan’s parent, KS2)

Three of the older participants held responsibility for self-management, using relaxation tools (‘I do the *headspace the APP. . . it does like calm you*’, Sophia, KS2), identifying low energy activities that they enjoyed (‘*for green* [low energy activities]. . . *sometimes I give my cat cuddles*’, Olivia, KS2) and using visual cue systems to regulate activity:

*I use a timer. Once the half hour goes you use the card and then every week you get to upgrade half. . .. And then you get more cards so more time, um, and that works a lot, but if I’m round my dad’s for example I still time how much I’ve done. I will just take it away from my red time.* (Willow, KS2)

Parents frequently felt that recommendations to limit activity and avoid overexertion was ‘*not appropriate for* [child’s] *age, needed to be adapted’* (Emma’s parents, KS1). Parents consistently expressed that children expend higher levels of physical and cognitive energy when well, that they ‘*move from one activity to the next*’ (Emma’s parents, KS1) and ‘*don’t lend themselves to quiet, low level activity’* (Ethan’s parent, KS2). Children reported ‘*it’s hard to find orange* [low level activity] *things to do*’ (Sophia, KS2). This meant that families found it ‘*challenging*’ or ‘*impossible*’ (Zoe’s parent, KS1) to monitor and restrict activities and frequently reported giving up on the activity diaries. Instead, families made macro adjustments to family life and structured the child’s environment to be conducive to sedentary behaviour, for example having ‘*pyjama days*’ (Sophia, KS2), where families stayed in the house rather than going-out or swapping physically exerting transport such as cycling and walking, for driving.

#### Theme 3: Parents responsible for managing condition, but unsupported in this role

The developmental capacities of KS1 children meant parents took full responsibility for implementing treatment ‘*to be honest it’s been me doing it’* (Emma’s parents, KS1). Parents still held much of the responsibility with KS2 children, but families talked about monitoring and regulating being more of a joint effort ‘*It’s kind of both of us*’ (Connor, KS2).

Some parents wished to protect a sense of a ‘normal’ childhood and expressed a desire to protect their child from the concept of having a chronic health condition. This was particularly true for parents of younger children. Parents were wary of their child being exposed to certain aspects of clinical discussions; they did not want their child upset by ‘*scary*’ (Connor’s parent, KS2) or ‘*negative*’ (Jacob’s parent, KS2) messages about CFS/ME. As such, some parents withheld information or refrained from asking certain questions in front of the child and felt that parent only sessions may be beneficial

*I think one-to-one time with me would have been really helpful because I felt I couldn’t say in front of [child] how worried I was. I didn’t want her to pick up on that. In fact in one of the appointments I wrote it down on a piece of paper and gave it to them and said um I’m not going to say it in front of [child] but please know that I am concerned.* (Emma’s parent, KS1)

Parents felt unsupported and ‘*very alone managing it*’ (Sophia’s parent, KS2). Parents consistently described the process of liaising with GPs and obtaining a referral to specialist services as a ‘*difficult journey*’ (Ethan’s parent, KS2), ‘*fighting a losing battle*’ (Jacob’s parent, KS2), feeling ‘*dumped*’ (Henry’s parent, KS1) and ‘*disbelieved*’ (Jacob’s parent, KS2). They described numerous visits to health professionals and protracted time to diagnosis and/or referral to a specialist service. Some also felt unsupported by the child’s school. Parents frequently talked about the value of the specialist service in alleviating the sense of isolation; they felt *‘relief that. . . somebody has listened’* (Jacob’s parent, KS2), ‘*believed*’ (Henry’s parent, KS1) and supported. Some parents wished there was more social support, facilitated by the service (‘*talking to people going through the same thing, another parent, would have been great’*, Ava’s parent, KS1).

## Discussion

Parents’ and children’s experiences showed that CFS/ME in younger children is disabling and complex. Parents expressed a desire for more in-depth explanations of this complex diagnosis, and for treatment to account for co-morbidities. There was important variation across the developmental range (from 5 to 11 years); the ability to engage in clinical consultations, describe experiences, understand diagnosis and self-manage increased with age. Parents highlighted the difficulties of regulating activity in younger children. Parents retained high responsibility for their child’s treatment but felt isolated and unsupported in this role.

### Strengths and limitations

Qualitative methods allowed us to gain a rich understanding of the views of children and caregivers. We interviewed 22 participants, gathering a diverse sample in terms of the child’s gender, illness severity and time of onset. We recruited from the largest specialist paediatric CFS/ME service in the UK, which offers a service to children across the UK due to a lack of local service provision (particularly for children under 12), and using Skype allowed us to include families from across the UK.

Findings from families engaged in specialist treatment may not be transferable to other families. We did not capture the views of black and minority ethnic groups. Few KS1 children chose to participate in the interview (*n* = 2), and the qualitative interview method, which required free-recall of past events and verbal fluency, posed a challenge for this age group. Although this limited the voices of the very young children it illustrated the problems of providing treatment for this age group.

The themes incorporate child and parental views. More parents than children participated and the power-dynamics of adults and children, could have potentially led to parents’ voices dominating the themes. A further issue is that parent-child power-dynamics in the dyadic interview may have limited the extent to which children felt able to express dissenting views. However, we worked hard to include the child’s voice by using art-based methods (Coad, 2007) and using dyadic interviews to increase the child’s confidence. At this younger age, children are likely to have close relationships with their parent and shared experiences, therefore, we believed that parents could provide prompts that would encourage richer accounts from the child.

### Implications in the context of literature

There are significant developmental advances from early to late childhood (Eccles, 1999), and this study indicates that interventions should not be delivered uniformly across this age group. In this study, children aged 5 to 7 years were less engaged in treatment and relied heavily on caregivers. This suggests that parent/carer-only sessions may be indicated for children aged seven and under, which is consistent with approaches used in other health services (Thirlwall et al., 2017). Parent/carer-only consultations may reduce the burden on the child (impact of appointments on fatigue, schooling, medicalisation), they can also be beneficial in enhancing parenting skills, increasing parents self-efficacy, providing more individualised and culturally-sensitive strategies (since the parent knows the child better than the clinician), and being more efficient (Leong et al., 2009; Salloum et al., 2014). However, this must be balanced with the child’s rights to be heard (United Nations Convention on the Rights of the Child (UNCRC), 1989), and the individual needs and wishes of the family should be considered when making decisions about parent-only sessions. Further research is needed within paediatric CFS/ME services to test the optimal model of parent-child involvement, investigating acceptability, efficacy and cost-effectiveness.

This study found that children over eight were increasingly engaged in treatment, consistent with the idea that this age group are ‘competent, independent, self-aware, and involved in the world beyond their families’ (Eccles, 1999), with developing reasoning and Illness beliefs (Erickson et al., 2005; Koopman et al., 2004). Therefore children aged 8-11 should be actively involved in treatment, with support from parents/carers. This includes helping children describe their experiences, understand their condition and its treatment, and engage in self-management. This study suggests that tools such as body mapping, emoji cards and card sorting may be effective in helping children over eight years describe their experiences of CFS/ME. In terms of providing developmentally appropriate explanations of diagnosis and treatment, there are examples of health education materials designed for this age group (e.g. from the fields of asthma, cystic fibrosis, leukaemia, and congenital heart disease), and these indicate that explanations should be ‘concrete and focused on behavioural recommendations’ (Erickson et al., 2005). Using play, arts, games and storytelling methods may also be appropriate for educating this age group (Brigden et al., 2019). With regards to promoting self-management, only a minority of the children in this study were using strategies to monitor and regulate their activity levels. However, children aged eight and upwards are typically involved in treatment (Brigden et al., 2019), they can be supported to self-manage their condition (e.g. self-administering insulin injections), and it is ethical and effective to involve children as much as possible (Alderson, 2017). This suggests that CFS/ME services need to develop innovative solutions to engage children aged eight and over in treatment. Integrating wearable technology into CFS/ME interventions may be a solution for supporting children to self-monitor and self-regulate activity and sleep behaviours. Wearable technology has the potential to feedback accurate data about a child’s health, or adherence to the intervention, without placing a burden on the parent or child to actively monitor behaviours. Wearable technology has been developed for a variety of paediatric conditions, including ADHD (Tavakoulnia et al., 2019), mood disorders (Sequeira et al., 2020), juvenile idiopathic arthritis (Heale et al., 2018), cerebral palsy (Bialocerkowski, 2011), asthma (Siering et al., 2019), and epilepsy (Bruno et al., 2018). Child-centred design principles could be incorporated, such as rewards and gamification (Brigden et al., 2020). Parents highlighted the challenges of keeping track of their child’s activity, and so the use of such wearable technology could also aid parents with monitoring and regulating their child’s behaviour. Integrating wearable technology into behavioural interventions for younger children is in its infancy, with little evidence regarding acceptability and efficacy. Furthermore, there are no existing wearable interventions specifically for children with CFS/ME, and so this would require further research to develop and test this.

Parents/carers reported a sense of isolation, consistent with previous studies (Mihelicova et al., 2016; Missen et al., 2012). Attending to the well-being of parents/carers caring for a child with a chronic health condition is important (Cousino & Hazen, 2013), and so interventions for younger children may benefit from attending to the support needs of parents/carers, given their increased responsibility and isolation. Offering parent-only sessions within the CFS/ME service may provide an opportunity for parents to talk about their own wellbeing, and the service could integrate brief interventions with parents/carers, to enhance their coping (Melnyk et al., 2001). The role of social support in coping is well documented (Ozbay et al., 2007), and parents/carers expressed a desire to meet with other families living with CFS/ME. The clinical service could facilitate this, for example, through sharing contact details or by running group sessions.

This study suggests how CFS/ME treatment approaches could be adapted for children under the age of eight years, for children aged eight to eleven years and for parents/carers. Given that this patient-group has been previously neglected in the research literature and given that parents/carers often feel isolated and excluded, it is particularly important that any service developments are carried out in collaboration with families. Co-design methods (Boden et al., 2018) could be used to ensure families are fully involved in service development, and this co-design process would be enhanced by involving other key stakeholders, such as clinicians.

## Supplemental Material

Appendix-_Topic_Guide – Supplemental material for Chronic fatigue syndrome/myalgic encephalomyelitis in children aged 5 to 11 years: A qualitative study Click here for additional data file. Supplemental material, Appendix-_Topic_Guide for Chronic fatigue syndrome/myalgic encephalomyelitis in children aged 5 to 11 years: A qualitative study by Amberly Brigden, Alison Shaw, Emma Anderson and Esther Crawley in Clinical Child Psychology and Psychiatry
