# Supplementary material for: Chronic fatigue syndrome/myalgic encephalomyelitis in children aged 5 to 11 years: A qualitative study
Source: Clin Child Psychol Psychiatry. 2020 Oct 22;26(1):18–32. doi: 10.1177/1359104520964528 (PMC7802053; doi:10.1177/1359104520964528)
Supplement: Appendix-_Topic_Guide – Supplemental material for Chronic fatigue syndrome/myalgic encephalomyelitis in children aged 5 to 11 years: A qualitative study [file Appendix-_Topic_Guide.pdf]

Topic Guide: Parents/ carers

This topic guide is for in-depth, semi-structured interviews with parents/carers to explore the following research questions:

- What are the parents/carers experiences / narratives of their child's CFS/ME?
- What are their views on CFS/ME treatment, and how it can be adapted for younger children?

Prior to start of the interview

- Check the participant has read the information sheet. Verbally go through information and answer any questions that the participant has.
- Complete consent forms.
- With consent, turn on audio recorder

Section 1: Narrative Protocol

- Start with opening question: "I want to learn about you and your child's story. Begin from around the time your child developed the condition and describe what has happened up until now"
  When the narration starts, it must not be interrupted until there is a clear ending, meaning that the interviewee pauses and signals the end of the story. During the narration, the interviewer abstains from any comment other than non-verbal signals of attentive listening and explicit encouragement to continue the narration. The interviewer may, however, take occasional notes for later questioning, if this does not interfere with the narration. Restrict yourself to active listening, non-verbal or paralinguistic support and showing interest ('Hmm, 'yes', 'I see'). While listening, develop, in your mind or on paper, the questions for the next phase of the interview. When the informant marks the end of the story, probe for anything else: 'is this all you want to tell me?' or 'is there anything else you want to say?'

  Prompting questions:

Section 2: Treatment

Note: the facilitator might also ask additional questions to clarify information/ use probes encourage the interviewee to say more about a particular topic.

- Start the section by saying "Now I would like to hear more about the treatments your child has had."
- Can you tell me about any treatment your child had before accessing the CFS/ME service?
- Can you tell me about the initial assessment appointment with the CFS/ME service? Prompting questions:

- How did you and your child explain your experience to the clinician?
- How did the clinician explain CFS/ME to you/ your child?
- How did the clinician explain what would happen next?
- What did you do after assessment?

- Can you talk to me about treatment with the service?
  Prompting questions:

  - Can you describe the treatment?
  - How was it described to your child?
  - Does your child understand about treatment?
  - Are you able to follow the treatment?
  - Anything that has worked well?
  - Anything that has made it difficult?

- Can you talk to me about ways you think the treatment could have been improved for a someone your child's age?

Section 3: Close

- Are there any issues that we have not talked about that you would like to raise?
- Clarify what happened next and who the participant can contact if they have any questions.
- Thank participants for their contribution.
